# Genotypic and Phenotypic Versatility of *Aspergillus flavus* during Maize Exploitation

**DOI:** 10.1371/journal.pone.0068735

**Published:** 2013-07-19

**Authors:** Massimo Reverberi, Marta Punelli, Valeria Scala, Marzia Scarpari, Paolo Uva, Wieslawa I. Mentzen, Andrea L. Dolezal, Charles Woloshuk, Flavia Pinzari, Anna A. Fabbri, Corrado Fanelli, Gary A. Payne

**Affiliations:** 1 Dipartimento di Biologia Ambientale, Università “La Sapienza”, Roma, Italy; 2 CRS4 Bioinformatica – Parco Scientifico e Tecnologico Polaris, Pula (CA), Italy; 3 Department of Plant Pathology, North Carolina State University, Raleigh, North Carolina, United States of America; 4 Department of Botany and Plant Pathology, Purdue University, West Lafayette, Indiana, United States of America; 5 Centro di Ricerca per lo Studio delle Relazioni tra Pianta e Suolo, CRA, Roma, Italy; University of Wisconsin – Madison, United States of America

## Abstract

*Aspergillus flavus* is a cosmopolitan fungus able to respond to external stimuli and to shift both its trophic behaviour and the production of secondary metabolites, including that of the carcinogen aflatoxin (AF). To better understand the adaptability of this fungus, we examined genetic and phenotypic responses within the fungus when grown under four conditions that mimic different ecological niches ranging from saprophytic growth to parasitism. Global transcription changes were observed in both primary and secondary metabolism in response to these conditions, particularly in secondary metabolism where transcription of nearly half of the predicted secondary metabolite clusters changed in response to the trophic states of the fungus. The greatest transcriptional change was found between saprophytic and parasitic growth, which resulted in expression changes in over 800 genes in *A. flavus*. The fungus also responded to growth conditions, putatively by adaptive changes in conidia, resulting in differences in their ability to utilize carbon sources. We also examined tolerance of *A. flavus* to oxidative stress and found that growth and secondary metabolism were altered in a superoxide dismutase (*sod*) mutant and an alkyl-hydroperoxide reductase (*ahp*) mutant of *A. flavus.* Data presented in this study show a multifaceted response of *A. flavus* to its environment and suggest that oxidative stress and secondary metabolism are important in the ecology of this fungus, notably in its interaction with host plant and in relation to changes in its lifestyle (i.e. saprobic to pathogenic).

## Introduction

Mycotoxin contamination of food and feed poses a serious concern for animal and human health. Aflatoxins, one of the major classes of mycotoxins, have received considerable attention, but research and regulatory efforts to mitigate the impact of aflatoxins have not resulted in satisfactory reduction of this carcinogen in the food supply. Control of this disease has been problematic, owing in part to our lack of understanding of the conditions that lead to infection and aflatoxin production by the fungus.

Prior to the 1970s aflatoxin contamination was thought to be only a post-harvest disease [1]. It is now recognized that pre-harvest aflatoxin contamination is a serious problem world-wide and the major source of contamination in developed countries. Therefore, effective control should ideally prevent contamination both in the field and in storage. This disease has been well studied and both saprophytic and parasitic phases of this fungus are important in its epidemiology [2]–[6]. As an example, the fungus survives in the soil as a saprophyte either on decaying tissues or as sclerotia, but it can be parasitic and invade developing kernels. Thus an understanding of both saprophytic and parasitic phases and of the factors which influence the switch between these phases, is important for sustainable control of this disease.

Several environmental signals are known to affect fungal morphology [7], [8], the formation of virulence factors [9] or toxic compounds [10], [11], and to induce the production of spores with different metabolic abilities [12]–[14]. Also, seed composition affects the ability of *A. flavus* to invade seed tissues [15]–[18]. Plant volatile organic compounds (VOCs), such as methyl salicylate and other oxylipins (e.g. green leaf volatiles), induce sporulation [19], [20] and modulate mycotoxins biosynthesis in several pathogenic fungi [21], apparently by replacing fungal endogenous oxylipins [21]–[24]. The discovery of an oxylipin-based cross-talk between maize and *A. flavus* may lead to new control strategies to limit AF contamination, such as the use of GM-maize with an altered oxylipin profile [25]. Further, the multiple and diverse factors crucial in controlling secondary metabolism in fungi have led the scientific community to adopt more holistic strategies for studying the implications of plant-pathogen cross-talk. By means of a global transcription analysis, it is possible to measure the expression of all the affected genes, including those regulated by pleiotropic effects. Recently, a whole-genome approach was applied to examine the expression of 56 secondary metabolism (sec-met) clusters in wild type and mutant strains for *A. flavus* grown under several cultural conditions [26]. The authors found the profile of sec-met cluster expression to differ among cultural conditions, including between parasitic and saprobic growth. Further, the authors found many of the sec-met clusters to be silent under all conditions examined. Silent clusters have been observed in other fungi, and many can be activated by chemical treatment, suggesting that these clusters may be active under certain ecological conditions [27].

Building on the observations by Georgianna et al [26] that ecological conditions can affect fungal gene expression, we examined both primary and secondary metabolite gene expression in *A. flavus* grown on substrates and under conditions that mimic four different trophic phases of the fungus. The four trophic environments were designed to mimic *in vitro* culture, saprophytic growth, initial contact with the host, and pathogenesis. We also examined carbon utilization of cultures started from conidia produced by *A. flavus* grown under the four trophic environments to determine if adaptation to a substrate impacts the metabolism of conidia produced on that substrate.

Several environmental signals are known to affect fungal morphology [7], [8], the formation of virulence factors [9] or toxic compounds [10], [11], and to induce the production of spores with different metabolic abilities [12]–[14]. Also, seed composition affects the ability of *A. flavus* to invade seed tissues [15]–[18]. There is evidence that the quantity and presumably the metabolic state of conidia is controlled by the effect of environmental factors acting during sporulation [28] by exploiting an adaptive advantage of phenotypic memory [29].

In this study, we used molecular, physiological and genomic approaches to study *A. flavus* interaction with maize notably evidencing how the shift – saprobic to pathogenic status – is driven by changes in: secondary metabolites clusters expression, responses to oxidative stress and carbon metabolism of conidia produced from cultures grown under these conditions.

## Results and Discussion

### Transcription profiles of *A. flavus* in four different nutrient conditions and pathway analysis

We analysed the transcriptome of *A. flavus* using a custom Affymetrix GeneChip microarray containing all the predicted *A. flavus* genes. Using this array, the transcription levels of 13548 genes were analyzed in three biological replicates with four different conditions: AF3357 grown on CD medium (*flask*), grown in flasks with CD medium containing injured maize kernels within a closed dialysis tube (*chemo*), grown on autoclaved maize kernels (*sapro*) and grown on ears in the field (*in vivo*). Gene expression was compared as follows: *chemo* vs *flask* and *in vivo* vs *sapro*. Statistically significant differences were assessed by Differential Expression using Distance Summary (DEDS) analysis [30]. 815 differentially expressed genes at a significance level of 0.01 were identified. Out of these, 9 were significant for the chemotrophic stage (*chemo* vs *flask*), and 806 were significant for the pathogenic stage (*in vivo* vs *sapro*). The most differentially expressed genes are shown in Table 1 (the complete list of 815 genes are in Table S1). A subset of genes identified as differentially expressed on DNA microarrays were also verified by RT-PCR (Table 1). Changes in expression of predefined biological pathways and gene sets were analyzed (Table 2 and S2). Gene sets were obtained from Gene Ontology (1085 GO terms covering 4903 genes) and InterPro annotations (1437 domains covering 4900 genes). The statistical significance of the pathway ‘activation’ has been assessed by random permutations (see Methods for details). The complete results of the pathway analysis are presented in supporting information (Table S2) whereas in Table 2 the significantly (p<0,01) altered pathways are presented both comparing *chemo* vs *flask* and *in vivo* vs *sapro* conditions. The most significant (p<0.001) up-regulated pathways during the *chemo* phase in *A. flavus* were those related to carbohydrate transport, pentose-phosphate shunt, phospholipid biosynthetic process, response to oxidative stress, L-arabinose metabolic and cell wall catabolic processes. In *chemo* phase, *A. flavus* down-regulated DNA replication, RNA processing and protein translation pathways. The carbohydrate transport pathway is up-regulated in the *chemo* phase compared to that of the fungus grown in *flask*. Homologs of two up-regulated genes in *A. flavus,* within this pathway, have been shown to be involved in *Ustilago maydis* and *Candida albicans* virulence. Notably, the Srt1 transporter allows *U. maydis* to directly utilize sucrose at the plant/fungus interface without extracellular hydrolysis and without the production of extracellular mono-saccharides known to elicit plant immune responses [31]. In *C. albicans*, *jen1* encodes an MFS-related transporter expressed in glucose-poor niches within the host, which may be important in the early stages of infection in humans [32]. In *A. flavus*, a similar MFS (1918.m01405_at) was also co-expressed with an uncharacterized fungal specific transcription factor (1918.m01406_at). Thus, even in the early stage of *A. flavus –* maize interaction, the up-regulation of this hypothetical mini-cluster related to sucrose transport could allow the fungus to exploit the scarce nutrients available on seed surface, favoring its virulence.

**Table 1:** List of top differentially expressed genes (FDR<0.01) in different phases of *A. flavus* 3357 growth.

Comparison	probe	gene function/protein	log_2_ratio	2^−^α^Ct^
*Chemo vs Flask*	2043.m00004_at	oxidoreductase, short-chain dehydrogenase reductase, pseudogene	0,4	
*Chemo vs Flask*	1918.m01406_at	Fungal specific transcription factor domain containing protein	5,4	6,5
*Chemo vs Flask*	2258.m00410_at	expressed protein-related	5,3	
*Chemo vs Flask*	1918.m01405_at	Major Facilitator Superfamily protein, membrane transporter	7,2	7,5
*Chemo vs Flask*	2911.m00243_at	kelch-domain protein, putative, cell polarity protein	−0,4	-0.1
*Chemo vs Flask*	2689.m00650_at	MED7 protein, transcriptional initiation	-1,0	−2,1
*Chemo vs Flask*	2258.m00669_at	GTP binding protein (Gtp1), putative	−0,7	−0,5
*Chemo vs Flask*	1918.m01221_at	Histidine acid phosphatase family protein	−2,1	−0,17
*Chemo vs Flask*	2634.m00339_at	hypothetical protein	−1,7	
*In vivo vs Sapro*	1918.m00961_at	Protein similar to CwfJ C-terminus 1 containing protein	0,8	
*In vivo vs Sapro*	1866.m00688_at	pectin methylesterase, putative	2,5	4,8
*In vivo vs Sapro*	2856.m00234_at	MSF multidrug transporter, putative	2,15	
*In vivo vs Sapro*	1569.m00043_at	G protein-coupled receptor alpha-related	1,93	6,49
*In vivo vs Sapro*	2856.m00485_at	Glycosyl-hydrolases family 16 protein	0,6	
*In vivo vs Sapro*	1866.m00614_at	Mismatched base pair and cruciform dna recognition protein, putative	2,75	
*In vivo vs Sapro*	2911.m00773_s_at	NADH oxidase, putative	1,5	
*In vivo vs Sapro*	1918.m01408_s_at	NAD dependent epimerase dehydratase family protein	3,9	
*In vivo vs Sapro*	1918.m00745_at	pH domain containing protein	0,5	
*In vivo vs Sapro*	2842.m00344_at	Glutathione S-transferase, C-terminal domain containing protein	0,4	3,26
*In vivo vs Sapro*	2856.m00371_at	alpha-glucosidase alpha-amylase, putative	−3,4	
*In vivo vs Sapro*	2368.m00161_at	TPR Domain containing protein	−0,3	
*In vivo vs Sapro*	2368.m00464_at	oxidoreductase, zinc-binding dehydrogenase family protein	−1,2	
*In vivo vs Sapro*	1569.m00528_at	oxidoreductase, short chain dehydrogenase reductase family protein	−6,6	−0,68
*In vivo vs Sapro*	1569.m00529_at	ABC-2 type transporter family protein	−5,7	
*In vivo vs Sapro*	2043.m00037_at	elastinolytic metalloproteinase Mep, putative	−3,4	
*In vivo vs Sapro*	2689.m00427_at	Gamma-glutamyltranspeptidase family protein	−0,8	
*In vivo vs Sapro*	2689.m00458_at	D-isomer specific 2-hydroxyacid dehydrogenase, NAD binding domain containing protein	−1,7	
*In vivo vs Sapro*	2689.m00291_at	phytase, putative 1866.m00203_at TRNA binding domain containing protein	−1,4	

In particular, the expression profile of the chemotrophic (*chemo*) phase has been compared with the gene profile expressed during the growth on basal medium (*flask*) and the whole gene expression of the fungus during the pathogenic phase, i.e. during the ear colonization (*in vivo)* was compared with the gene expression of *A. flavus* during the growth on dead maize kernels (*sapro*). Gene expression changes were calculated by both log2 of microarray expression ratio (log_2_ ratio), and, for a chosen subset of genes (indicated in bold characters), as relative expression by a SYBR Green Real Time PCR approach.

**Table 2:** Biological processes from Gene Ontology (GO-BP) collection that are differentially expressed in *chemo* vs *flask* or *in vivo* vs *sapro* comparisons.

			Chemo vs Flask		In-vivo vs Sapro	
SETID	GO-Term	# genes	Chemo	p-value	In-vivo	p-value
GO:0005975	carbohydrate metabolic process	172	Up	0	Down	0
GO:0055114	Oxidation reduction	588	Up	0,0015	Down	0,0145
GO:0008643	carbohydrate transport	51	Up	0		
GO:0006098	pentose-phosphate shunt	13	Up	0,001		
GO:0008654	phospholipid biosynthetic process	19	Up	0,0035		
GO:0006979	response to oxidative stress	9	Up	0,0195		
GO:0006164	purine nucleotide biosynthetic process	5	Down	0,024	Down	0,004
GO:0009073	aromatic amino acid family biosynthetic process	10	Down	0,0105	Down	0,0215
GO:0000059	protein import into nucleus, docking	7	Down	0,009		
GO:0006807	nitrogen compound metabolic process	25	Down	0,0015		
GO:0006270	DNA replication initiation	6	Down	0,0125		
GO:0009116	nucleoside metabolic process	9	Down	0	Down	0,0245
GO:0015931	nucleobase, nucleoside, nucleotide and nucleic acid transport	12	Down	0,0005	Down	0,049
GO:0006412	translation	130	Down	0		
GO:0006886	intracellular protein transport	35	Down	0,0115		
GO:0006813	potassium ion transport	4	Down	0,0095		
GO:0008033	tRNA processing	8	Down	0,0095		
GO:0006397	mRNA processing	22	Down	0,005		
GO:0006511	ubiquitin-dependent protein catabolic process	34	Down	0,0025		
GO:0009086	methionine biosynthetic process	5			Down	0,0005
GO:0006508	proteolysis	131			Down	0,0005
GO:0009072	aromatic amino acid family metabolic process	11			Down	0,0025
GO:0008152	metabolic process	614			Down	0
GO:0006559	L-phenylalanine catabolic process	4			Down	0,011
GO:0009228	thiamin biosynthetic process	4			Down	0,012
GO:0006730	one-carbon compound metabolic process	4			Down	0,012

In the pathogenic phase, many pathways were significantly altered. In particular, genes for the ion transport, polygalacturonase activity, and aflatoxin biosynthesis were up-regulated whereas genes in the aromatic amino acid family of metabolic processes, and genes for ABC-transporters, hydrolases and cellulases were down-regulated (Table 2). After host recognition the fungus invades the endosperm and the lipid-rich embryo tissues of developing maize kernels [5]. In the comparison between living (*in vivo*) and dead kernels (*sapro*), we found 806 differentially expressed genes. Clearly, the living tissue of kernels thoroughly modulates gene expression in the fungus. Many of the biosynthetic pathways related to fungal primary metabolism are down-regulated *in vivo* phase (i.e. nitrogen compounds biosynthetic processes, protein synthesis, expression of ABC transporters and α-amylase).

As expected, lipid metabolism is up-regulated in pathogenesis since lipids are known to be involved in plant-pathogen communication and recognition. Lipids and oxylipins represent one of the most effective signals for controlling pathogen morphogenesis, development and virulence by altering the host oxylipins pathway [21], [23], [33]. Recently, it was reported that *A. flavus* employs a lipid-based cross-talk with its host [33] through G protein coupled receptors (GPCRs) [34]. We found that at least one member of this receptor family is strongly up-regulated in the *in vivo* phase. This gene (1569.m00043_at) shares a strong sequence identity with a Pth11p-like protein (acc. n. XP_002379256, score 151, max id. 72%), a cell-surface integral membrane protein required for pathogenicity in several fungi [32]. Further studies are presently under way for individuating the host signals that switch on the expression of this GPCR.

### Biolog Phenotype MicroArray (PM) analysis

**Figure 1:**
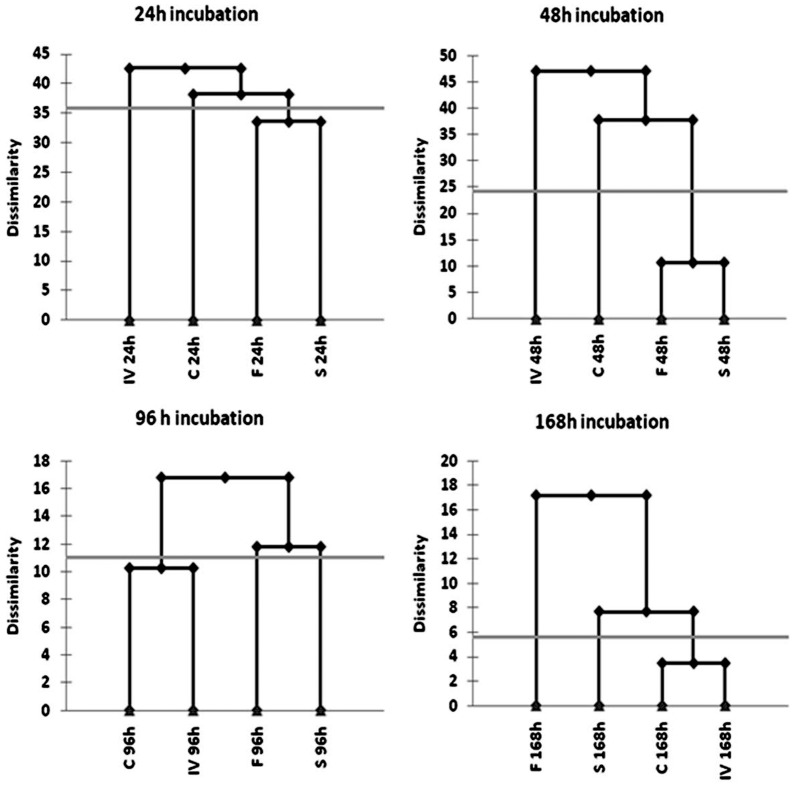
Hierarchical clustering based on the carbon source utilization profiles after 24, 48, 96 and 168 h of incubation of cultures started from conidia isolated from the four trophic phases (*in vivo, sapro, flask* and *chemo*). The gray horizontal lines indicate distance measures of the carbon source utilization profiles and describe the metabolic similarity between the phases at different incubation intervals. The dendrograms show the progressive grouping of the data (and therefore the higher or lower similarity between trophic phases) along the microplates incubation period (24, 48, 96 and 168 h).

Cultures started from conidia of *A. flavus* harvested from the four different trophic phases (*chemo, flask, sapro, in vivo*) and incubated on 95 different carbon sources show marked differences in growth. Figure 1, shows Agglomerative Hierarchical Clustering (AHC) of the four trophic phases, based on Euclidean distance measures of the carbon source utilization profiles from 24 to 168 h of incubation. AHC describes the metabolic similarity between the phases at different incubation intervals. The resulting dendrograms show the progressive grouping of the data (and therefore the higher or lower similarity between trophic phases) along the microplates incubation period. The four trophic phases (*in vivo, sapro, flask* and *chemo*) are always separated in different clusters along the whole microplate incubation period. This result suggests that the past culture history of a fungal spore produces some kind of adaptive memory in the following generation possibly due to accumulated molecules and transcripts or, in a more suggestive hypothesis, in a sort of trans-generational epigenetic inheritance [35]–[37] that deserves extensive histone chromatin assays to be better evaluated. Greater distances can be observed between the four trophic phases at 48 and 96 h of incubation. Cultures started from conidia produced in the *in vivo* treatment show a strongly different behavior on Biolog plates substrates compared to conidia harvested from the other three treatments; anyway, after 96 h of incubation the growth and substrate use appears metabolically similar to that exhibited by the conidia coming from the *chemo* condition. The metabolic distance between the *in vivo* and the *sapro* cultures is high over the whole incubation period (Euclidean distance, Ward's agglomeration method with a truncation for 0.001<p<0.05). Interestingly, cultures started from conidia produced in the *chemo* and the *in vivo* phases appear to cluster more closely at all-time point.

A more detailed evidence of a different metabolic behaviour of conidia with a different past culture history is shown in Tables 3 and 4 where the 95 substrates were divided into 15 categories plus water in accordance with Atasanova & Druzhinina [38] and the average absorbance (OD_490_ readings indicating the metabolic rate – see Methods section) for all wells in each category calculated. Table 3 reports data after 48 h and Table 4 data after 96 h of incubation. The conidia from the four different growth phases use the substrate categories at a significantly different degree: the black/white gradient in the tables, graphically represents the degree of overall use of group-substrate, and shows that C =  *chemo*, and F =  *flask* phases are much more efficient in substrate use than IV =  *in vivo* and S =  *sapro* phases. Conidia produced in the latter appear particularly inefficient in the use of “Biogenic and heterocyclic amines”, “Heptoses”, “Hexosamines” and “Polyols” categories of substrates, when compared with the conidia from *chemo* and *flask* phases. The sugars with seven carbons (Heptoses) are used mainly in the *flask* phase, while “Glucosides” are metabolised in a markedly different way by the four phases. Conidia from the *sapro* phase metabolise Polyols and L-aminoacids faster than the other substrate categories, while Peptides are more used in the *flask* phase. The differences observed between the phases according to the 15 categories of substrates are present at both 48 and 96 hours of incubation, but with the rise of incubation time some differences between the phases, in terms of substrate use, become less significant, thus suggesting that for some categories of compounds only the speed is different, but not the overall ability to use it as sole carbon source.

**Table 3:** One-way ANOVA for Time 48-hours incubation.

	C		F		IV		S	
Water	0.642	B	0.375	AB	0.206	A	0.223	A
Heptoses	1.436	B	0.470	A	0.248	A	0.255	A
Hexoses	1.130	B	1.089	B	0.515	A	0.495	A
Pentoses	0.939	B	1.116	B	0.415	A	0.490	A
Sugar acids	1.152	BC	1.540	C	0.795	AB	0.582	A
Hexosamines	1.571	C	0.926	B	0.472	A	0.379	A
Polyols	1.428	B	1.598	B	0.578	A	0.530	A
Polysaccharides	1.104	C	0.834	BC	0.432	A	0.568	AB
Oligosaccharides	1.221	B	0.999	B	0.357	A	0.424	A
Glucosides	1.345	B	1.242	B	0.466	A	0.438	A
Peptides	1.139	BC	1.434	C	0.952	AB	0.661	A
L-amino acids	1.172	B	1.494	C	0.926	AB	0.670	A
Biogenic and heterocyclic amines	1.046	C	1.225	C	0.479	B	0.110	A
TCA-cycle intermediates	1.178	B	1.214	B	0.921	B	0.293	A
Aliphatic organic acids	0.785	B	0.523	AB	0.646	AB	0.219	A
Others	0.926	B	0.976	B	0.478	A	0.382	A

The ANOVA, followed by Tukey's HSD t-test, was run on respiration values (OD_490_ readings) after 48 h of incubation. Statistically significant differences (p<0.001) in substrate use between the growth phases (C* =  chemo,* F* =  flask,* IV* =  in vivo,* S* =  sapro*) are marked with different letters (A, B, C, D).

**Table 4:** One-way ANOVA for Time 96-hours incubation.

	C		F		IV		S	
water	0.624	B	0.540	B	0.154	A	0.190	A
Heptoses	1.475	C	0.650	B	0.213	A	0.203	A
Hexoses	1.787	C	1.642	C	1.255	B	1.055	A
Pentoses	1.592	B	1.483	B	1.134	A	0.983	A
Sugar acids	1.278	B	1.606	C	0.882	A	1.043	A
Hexosamines	1.715	C	1.048	B	0.768	A	0.763	A
Polyols	2.357	B	2.344	B	1.545	A	1.370	A
Polysaccharides	1.485	C	1.031	B	0.889	AB	0.802	A
Oligosaccharides	2.055	C	1.406	B	1.198	AB	0.993	A
Glucosides	2.211	D	1.758	C	1.370	B	1.076	A
Peptides	1.378	B	1.920	C	1.347	B	1.097	A
L-amino acids	1.537	C	1.752	C	1.021	A	1.271	B
Biogenic and heterocyclic amines	1.273	C	1.676	D	0.776	B	0.549	A
TCA-cycle intermediates	1.325	B	1.700	C	1.009	B	0.523	A
Aliphatic organic acids	1.473	C	1.199	BC	0.971	B	0.286	A
Others	1.408	B	1.442	B	0.779	A	0.791	A

The ANOVA, followed by Tukey's HSD t-test, was run on respiration values (OD_490_ readings) after 96 hours of incubation. Statistically significant differences (p<0.001) in substrate use between the growth phases (C* =  chemo,* F* =  flask,* IV* =  in vivo,* S* =  sapro*) are marked with different letters (A, B, C, D).

**Figure 2:**
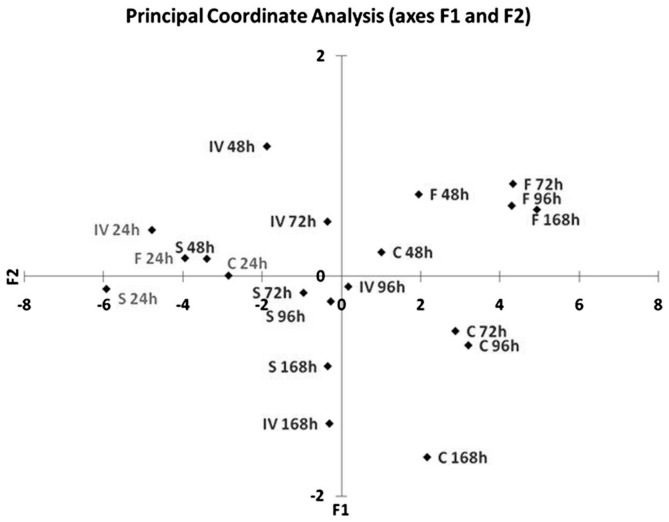
Scatter plot of the first two principal components from the Principal Coordinate Analysis of the carbon source utilization profiles of conidia conidia isolated from the four trophic phases (*in vivo-*IV, *sapro-*S, *flask*-F and *chemo-* **C) after 24, 48, 72, 96 and 168** h of incubation.

The dissimilarity between the groups is summarized and visualized in Figure 2, where the plot obtained with Principal Coordinate Analysis is reported. The mycelia derived from the conidia harvested at completion of the four trophic phases are similar in their metabolism after 24 h of incubation (they are all reported in the same quadrant of the plot). The metabolic behaviour of the different trophic phases diverges with higher incubation times: the *flask* phase (F) is represented in the positive quadrant (top right), while conidia from *chemo* (C) phase are in the bottom right quadrant. Both *in vivo* (IV) and *sapro* (S) phases are on the left side of the plot and show some overlap at longer incubation times.

The ANOVAs analyses (Table S3 in supporting information), performed on single substrates, show different groupings according to the incubation times. Some substrates are more significant in separating the trophic phases than others, indicating that a thorough study should be considered to create a pool of indicative substrates that can better address the fitness of spores in pathogenic species, and help in predicting the ecological fitness of *A. flavus*. Interestingly, the control medium (water) used in this study is also able to distinguish behavioral differences between cultures started from conidia obtained from the different trophic studies. Conidia from the *in vivo* and *sapro* treatments do not germinate as well as those from the *chemo* and *flask* conditions (ANOVA, Tukey's HSD test with significance of the differences for p<0.001). Although all fungal spores presumably contain abundant potential metabolic substrates, their metabolic activity is expressed only when their dormancy is broken. Dormancy and the induction of germination are under specific biochemical control in yeast spores [13], [14]. The maintenance of dormancy seems to be caused by ATP feedback-inhibition of the low-activity form of the enzyme trehalase. Activated trehalase triggers the mobilization of trehalose during early germination. The ANOVA results at 96 h of incubation for trehalose utilisation by the different trophic phases show the same grouping obtained for water, indicating that some of the main differences between the metabolic “aptitudes” of the conidia, coming from the different phases, can be related to the ability or the speed of germination at a given condition (Table S3).

### Promoter sequence analysis

The promoter regions were searched for the presence of the motifs TGACTCA recognized by the yeast oxidative stress-related transcription factor AP-1 (and putatively by its orthologous AfyapA) and TCGn{5}CGA, the binding site for the aflatoxin regulator, aflR. In fact, oxidative stress and AF synthesis are closely related as demonstrated by the presence in *Aspergillus* cell of the oxidative-stress transcription factor Ap*yapA* which “senses” the oxidative stress into the cytoplasm and, by transcriptional control together with AftB, activates, ultimately, antioxidant enzymes to scavenge the excess of oxidants [39]–[41]. In fact, the reduction of the cell environment leads to the control/inhibition of AF synthesis [42]–[44]. Of the 806 differentially expressed genes in the *in vivo* vs *sapro* comparison, the AP-1 motif was more enriched in the down-regulated genes (78 out of 472 genes), compared to its frequency (1549) in the genome (p<0.001). In relation to these results, the behavior of strains of *A. flavus* disrupted in redox regulating genes has been investigated below.

### Response to oxidative stress

The transcription profile indicates that gene activation in response to oxidative stress conditions may be necessary for growth during the *chemo* phase. In relation to this, differential expression of genes in pathways in the *chemo* phase is associated with metabolic processes, such as the pentose pathway, and oxidation/reduction pathways, leading to a shift toward secondary metabolism.. To further study the crucial role of oxidative stress in fungal growth, metabolism and plant-fungus interaction, we use two mutant strains of *A. flavus* in which a copy of a Mn-superoxide dismutase (AFLA_033420) and of an alkyl hydroperoxide reductase (AFLA_019280) genes have been disrupted. The enzymes coded by these genes scavenge superoxide anions and peroxides in the cell to prevent the excessive accumulation of these dangerous oxidants. These mutant strains are grown in the same four trophic conditions and compared to the WT strain (i.e. *flask, chemo*, *sapro* and *in vivo*) for the growth rate and the biosynthesis of aflatoxins (Table 5). The two mutants grow slower than the WT strain in all the culture conditions. The AF biosynthesis in *flask, chemo* and *sapro* phase is significantly higher in the mutant strain Δ*ahp* in comparison to the WT and the Δ*sod* strain. Furthermore, both mutant strains synthesize a significantly lower amount of AF in the *in vivo* phase (Table 5). In general, the unexpected lack of increase in aflatoxin biosynthesis in these redox-altered mutants may be related to the lowering of their growth rate. Specifically, the reduction in superoxide anion scavenging efficiency, profoundly affects *A. flavus* growth. We can hypothesize that the ROS produced in the *A. flavus-*maize interface can significantly affect Δ*sod* fitness, impairing its ability to synthesize toxins. In relation to this, Δ*sod* mutant treated with cumene hydroperoxide (CH, 1mM), an oxidative stressor, grows similarly and produces less aflatoxin compared to the WT strain (Figure S1). Moreover, when an inactivation of one copy of the mitochondrial [45] Mn-dependent *sod* gene occurs, ROS accumulation can affect the energy conservation by inactivating the enzyme aconitase subsequently inhibiting both the Krebs cycle [46] and the electron flux in the mitochondria with the increase of its internal membrane permeability [47]. This mitochondria malfunction probably causes a scarce uptake of nutrients and a reduction in the growth rate. In this condition, Δ*sod* may address its energy mainly for maintaining the growth rate and the residual antioxidant defences, rather than synthesising AF (“luxury molecules”). The relationship between growth and aflatoxin production in Δ*ahp* strain appears to be more complex. Perhaps, this is related to the type of reactive species present on the seed surface. Maize seeds produces lipid hydroperoxide for hampering *A. flavus* growth [21], [25]. In fact, alkyl hydroperoxide reductases may also scavenge lipid hydroperoxides [48], [49]. Thus, maize seeds may react to *A. flavus* invasion by producing these reactive species and the Δ*ahp* strain is not “equipped” to efficiently react to the toxic effect of these hydroperoxides. In these conditions, we may expect that the Δ*ahp* strain too, drives its resources for surviving in an oxidative-hostile micro-environment rather than activating secondary metabolism.

**Table 5:** Analysis of fungal growth (mg/mL dry weight, d.w. or as ng fungal DNA/g maize seeds) and AFB1 synthesis (ng/mL) of WT, Δsod and Δahp strains of *A. flavus* disrupted in a Mn-dependent superoxide dismutase (acc. N. AFLA033420) or in an alkyl hydroperoxide reductase (acc. N. AFLA019280) respectively, grown for different time intervals (3–7 d) in *flask*, *chemo*, *sapro* and *in vivo* phases as described above.

	Fungal growth											
	*Flask* (mg d.w./mL)			*Chemo* (mg d.w./mL)			*Sapro* (ng DNA/g maize seeds)			*in vivo* (ng DNA/g maize seeds)		
	WT	Δsod	Δahp	WT	Δsod	Δahp	WT	Δsod	Δahp	WT	Δsod	Δahp
3d	2.1±0.1	1.9±0.2	1.8±0.5	2.5±0.3	2.0±0.2	1.9±0.1	1.5±0.1	0.7±0.3	0.7±0.2	1.0±0.5	0.5±0.1	0.4±0.1
4d	2.6±0.3	2.5±0.6	2.4±0.3	2.9±0.1	2.2±0.4	2.2±0.3	15.1±2.5	3.2±0.6	2.5±0.3	12±0.3	2.5±0.5	2.1±0.3
5d	2.7±0.5	2.6±1.0	2.5±0.2	3.0±1.0	2.5±0.7	2.3±0.2	18.2±3.5	6.5±0.9	3.7±0.5	15±2.2	5.1±0.9	4.5±0.6
6d	3.5±0.1	2.9±0.3	2.8±0.9	3.8±0.5	2.7±0.6	2.5±0.4	21.3±1.1	15.8±3.3	8.5±1.5	18±1.5	15.2±2.1	7.1±0.5
7d	3.9±0.8	2.9±0.1	2.9±0.2	4.2±0.3	2.8±0.1	2.7±0.5	32.6±2.2	21.2±4.1	13.5±2.2	25±3.2	20.3±1.5	12.2±2.2

	AFB1											
	*Flask* (ng/mL)			*Chemo* (ng/ml)			*Sapro* (αg/g maize seeds)			*in vivo* (αg/g maize seeds)		
	WT	Δsod	Δahp	WT	Δsod	Δahp	WT	Δsod	Δahp	WT	Δsod	Δahp
3d	9.1±0.8	1.0±0.3	21.1±1.1	6.5±2.1	1.1±0.2	18.2±0.9	0.1±0.1	0.1±0.1	1.1±0.3	0.1±0.1	0.005±0.001	0.1±0.1
4d	10.2±1.5	1.5±0.3	25.3±2.5	8.5±0.8	1.6±0.3	22.3±2.5	0.3±0.1	0.2±0.1	1.5±0.3	0.5±0.3	0.01±0.005	0.3±0.1
5d	12.2±2.2	2.1±0.5	24.9±3.3	9.2±0.3	2.2±0.3	24.2±3.2	1.1±0.2	0.5±0.3	2.5±0.5	1.8±0.5	0.5±0.1	1.1±0.3
6d	15.1±1.1	3.3±0.1	32.2±2.1	12.1±1.5	3.5±0.8	29.5±4.4	6.5±0.5	1.5±0.3	12.5±1.5	10.2±1.5	2.1±0.3	6.5±0.8
7d	25.2±3.2	3.5±0.6	65.1±7.5	19.5±2.5	3.6±0.6	41.2±5.2	10.2±0.8	3.2±0.2	20.5±1.8	16.1±2.3	4.5±0.5	9.3±1.5

The results are expressed as mean values (± SE) of a total of 6 independent replicates.

### Secondary metabolites cluster analysis

Aflatoxins are not the sole secondary metabolites produced by *A. flavus* as demonstrated by Georgianna and Payne [26] through the use of SMURF (Secondary Metabolite Unknown Regions Finder) software [50]. The SMURF program searches for genes encoding multifunctional enzymes (called backbone genes by SMURF) associated with non-ribosomal peptide synthetases (NRPSs) for non-ribosomal peptides, polyketide synthases (PKSs) for polyketides, hybrid NRPS–PKS enzymes for hybrids and prenyl-transferases (PTRs) for terpenoids. This software allows the transcriptional analysis of 56 individual secondary metabolite clusters within the genome of *A. flavus* [26], [51]. In our experiments, out of 56 secondary metabolites clusters identified, 24 were significantly (p<0.05) differentially expressed. Many of these secondary metabolite clusters are not yet annotated, thus it is not possible to assign them a specific biological role in the pathogenic process. Nevertheless, clusters 1 and 23 encoding polyketide synthases, clusters 11 and 31 encoding non-ribosomal peptide synthases and the aflatrem cluster were up-regulated in the *chemo* conditions. In contrast, the dimethylallyl tryptophan synthase cluster 19, the palmytoil-transferase cluster 16, NRPS-like cluster 35 [52] and clusters 4 encoding non-ribosomal peptide synthases, cluster 10 encoding the pigment formation (Arp1) and the AF gene cluster were down-regulated (Table 6).

**Table 6:** Secondary metabolite (sec-met) clusters which are differentially expressed in *chemo* vs *flask* or *in vivo* vs *sapro* comparisons.

				Chemo vs Flask		In vivo vs Sapro	
Collection	Sec-Met cluster ID	Backbone gene	# genes	Chem	p-value	In-vivo	p-value
Sec-met	11	Nonribosomal peptide synthetase-like enzyme, putative	11	Up	0	Up	0
Sec-met	1	Polyketide synthase, putative	9	Up	0.0015		
Sec-met	15	Dimethylallyl tryptophan synthase, putative	12	Up	0.002		
Sec-met	31		14	Up	0.0075	Down	0.0035
Sec-met	34		8	Up	0.034		
Sec-met	23	Polyketide synthase, putative	17	Up	0.041	Down	0
Sec-met	50		7			Down	0
Sec-met	21	amino acid adenylation domain containing protein	36			Down	0.024
Sec-met	42	Polyketide synthase, putative	11			Down	0.01
Sec-met	48	Nonribosomal peptide synthetase-like enzyme, putative	20			Down	0
Sec-met	8	Polyketide synthase, putative	9			Down	0
Sec-met	55	Dimethylallyl tryptophan synthase, putative	4			Up	0.011
Sec-met	47	Nonribosomal peptide synthetase-like enzyme, putative	5			Up	0.005
Sec-met	32		19			Up	0
Sec-met	5	Polyketide synthase PksP	3			Down	0.001
Sec-met	37	Nonribosomal peptide synthetase-like enzyme, putative	3			Down	0.0235
Sec-met	45	Nonribosomal peptide synthetase-like enzyme, putative	16			Up	0.0005
Sec-met	22	Nonribosomal peptide synthetase, putative	6			Down	0.0275
Sec-met	16		2	Down	0.0495		
Sec-met	19	Dimethylallyl tryptophan synthase, putative	6	Down	0.0205		
Sec-met	4	Nonribosomal peptide synthetase, putative	4	Down	0.025		
Sec-met	35		5	Down	0.0145		
Sec-met	54	aflC/pksA/pksL1/polyketide synthase	30	Down	0	Up	0
Sec-met	10		3	Down	0.0005		

We also investigate clusters that are differentially regulated in *A. flavus* during its interaction with maize (*in vivo* vs *sapro* comparison). Clusters 11, 32, 45, 47, 54 (aflatoxins), and 55 (CPA-cyclopiazonic acid) result strongly up-regulated. The ETP cluster, encoding for a fungal class of toxins (EpipolyThiodioxoPiperazines), as well as other sec-met clusters related to polyketide synthase metabolites and nonribosomal peptide synthetases are significantly down-regulated (Table 6). These data are consistent with results obtained by HPLC analysis of AF synthesis (see WT in Table 5). Some of the secondary metabolite clusters, that are up-regulated (clusters 11, 32, 45, 47, 54 and 55) *in vivo,* likely have a role in pathogenicity, development, or in the production of ecologically important compounds. For example expression of *nepA* (which is co-expressed with cluster 32) whose homologs in other pathogens are known pathogenicity factors [53], is up-regulated in the *in vivo* condition.

### Identification of groups of co-expressed neighbor genes: application to the secondary metabolite clusters

**Figure 3:**
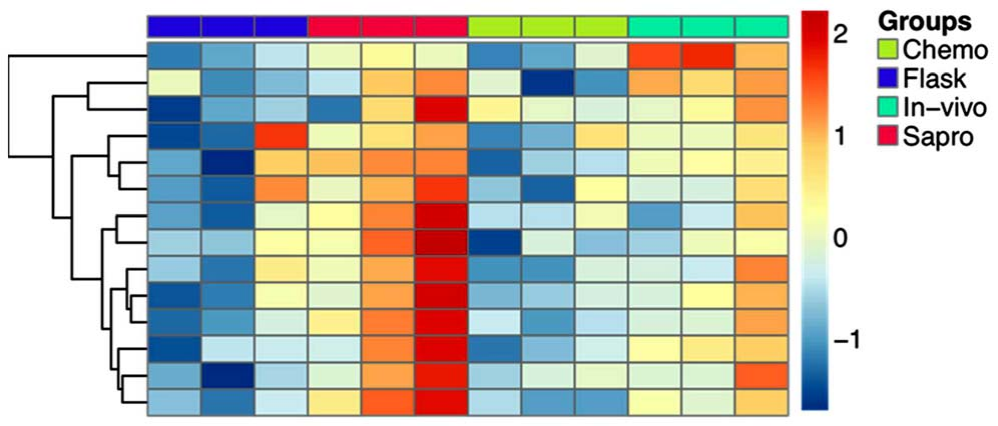
Expression profiles of the 14 genes in the ETP (epipolythiodioxopiperazines) secondary metabolite gene cluster. Relative expression values of individual replicates are shown. The expression level of each gene was normalized across all samples and is represented by a color whose intensity corresponds to the magnitude of deviation (σ) from the mean. The color-coded scale for normalized expression values maps is located at the right of the figure. *Flask,* replicates for *A. flavus* 3357 strain grown on CD medium; *sapro,* replicates for the *A. flavus* 3357 strain grown on dead maize kernels; *chemo,* replicates for the *A. flavus* 3357 strain grown in presence of injured viable maize kernels into the flask; *in vivo,* replicates for the *A. flavus* 3357 strain grown on the kernels into ears on the plant.

In general, the genes encoding pathway enzymes are highly co-expressed compared to genes selected at random from an entire genome. Thus, an analysis of gene co-expression performed from a microarray investigation allows one to predict putative members of certain pathways, either as new members, regulators, or consumers of pathway end-products. An example is given for the ETP cluster (Figure 3).

**Figure 4:**
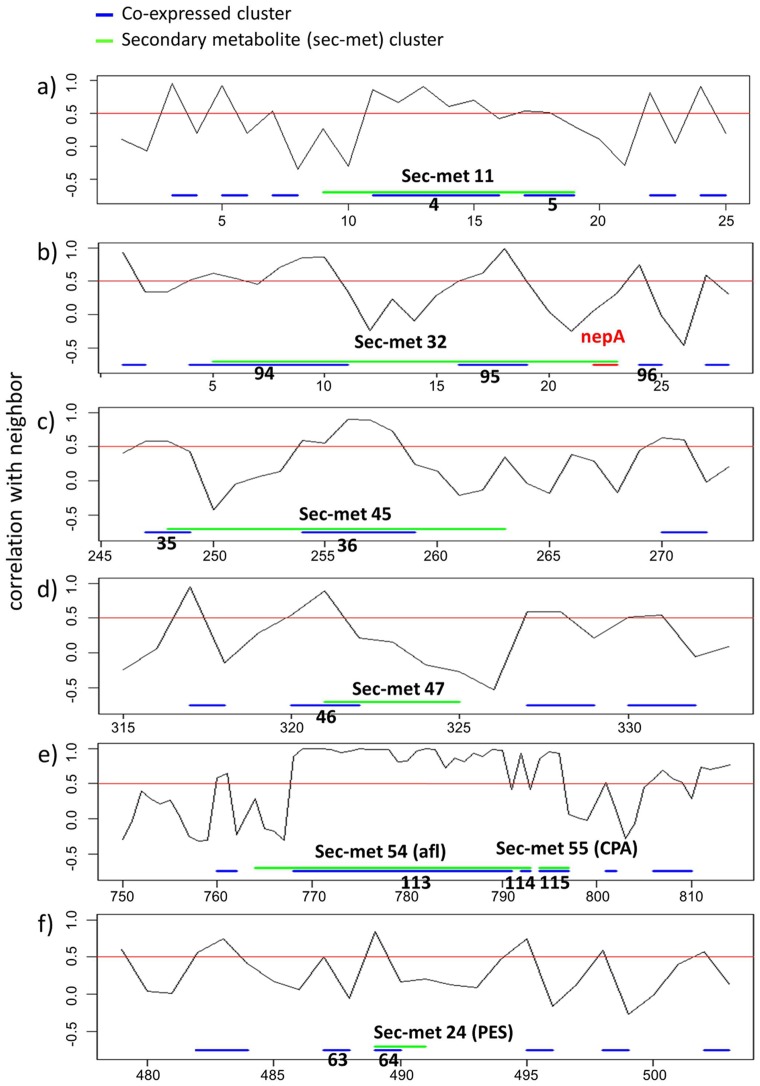
Plot of the local co-expression values (i.e. correlation between the expression of the neighboring genes). Groups of neighboring genes with correlation exceeding a threshold of 0.5 (marked by a red line) are identified as co-expressed clusters. Larger co-expressed clusters, with at least five members, are denoted by blue bars. Secondary metabolite clusters from Georgianna et al. (2010), identified with SMURF algorithm, are denoted by green bars. The *nepA* gene is indicated, inside the cluster 32, by red bar. The numbers on x axis indicate gene indexes along the contig. The individual panels are described in the Results section.

The application of our algorithm (see methods for details) to the combined microarray data set containing the 4 conditions tested in our study and the 28 experimental conditions from Georgianna et al. [11], has led to the identification of other genes likely clustered/co-regulated with previously described secondary metabolites gene clusters. The co-expression analysis has been performed for all the secondary metabolite clusters. Here we focus on those putatively involved in *A. flavus* pathogenesis, which are characterized by higher expression in *in vivo* as compared to *sapro* (clusters 11, 32, 45, 47, 54 and 55) (Figure 4A–F). Sec-met cluster 11, encoding non-ribosomal peptide synthetase, contains two subgroups of co-expressed neighbor genes covering 9 out of 11 total genes in the cluster (Figure 4A). The cluster 32, including the necrosis and ethylene-inducing peptide nepA, contains two subgroups of co-expressed adjacent genes (Figure 4B). The higher expression in *in vivo* vs *sapro* is driven by the subgroup 94, which is also correlated to the expression of the non-adjacent nepA gene. In particular, the probes most correlated (Pearson's r>0.5) to nepA are 2541.m00047_at (HAD superfamily hydrolase, putative) and 2541.m00046_at (conserved hypothetical protein). For the two clusters 45 and 47 (Figure 4C–D), we observe that only a fraction of the genes are co-expressed. However, in both cases we are able to identify additional co-expressed genes not identified by the SMURF algorithm (Table S4 in supporting information). The same co-expression analysis was carried out on the AF and CPA clusters (Figure 4E). Almost all the genes in the AF cluster (54) are co-expressed. A strong co-expression is also observed for all the genes in the CPA cluster (55), which is located immediately distal to the AF cluster. Recently, Georgianna et al. (2010) [11] observed a shared regulation for the four genes in the CPA cluster, thus supporting the hypothesis that they are part of a gene cluster that is necessary for the CPA biosynthesis. By co-expression analysis, we confirm the strong co-regulation of the genes in CPA cluster and we also observe a positive correlation with almost all the genes in the AF cluster. Our data suggest the existence of a common mechanism of regulation for the synthesis of both metabolites and thus, probably, of a putative common ecological role at least during the pathogenic process. We also applied the algorithm to the PES (Non-ribosomal peptide synthetase) cluster that is putatively involved with *A. flavus* pathogenesis. In fact, in *A. fumigatus,* a gene in this cluster, *pes1,* improved fungal tolerance against oxidative stress, during the infection process [54]. In the PES cluster (24), 2 out of the 3 genes are strongly co-expressed: a putative ABC multidrug transporter (2368.m00221_at) and the non ribosomal peptide synthase Pes1 (co-expressed cluster 64; Figure 4F). However, on average there is only a mild increase of the expression of the PES cluster members *in vivo* compared to *sapro* (t-test p-value  = 0.09).

Upstream of the PES cluster, we detect another pair of co-expressed adjacent genes (co-expression group 63) composed by the DNA repair protein RAD51 (2368.m00223) and a conserved hypothetical protein (2368.m00222).

## Conclusions

Gene expression profiling provides a systematic approach to dissect complex process such as the interaction of organisms with their environment. We used this approach, along with physiological studies, to better characterize the transition of *A. flavus* from saprobic growth to invasive pathogenic colonization of living maize kernels. *A. flavus* is a fungus that is ecologically competitive as a saprobe on several substrates and as a pathogen of plants and immunocompromised humans [4], [5], [15], [17], [55]. In our study, we found transcriptional changes in both primary and secondary metabolism genes depending on the substrate colonized. Pathogenic growth of the fungus in living kernels led to a change in expression of 806 genes compared to saprobic growth. Notable changes in this trophic shift included a change in expression of several genes encoding secondary metabolites. These included higher expression of the aflatoxin biosynthetic cluster, and clusters for two other mycotoxins, cyclopiazonic acid and aflatrem. Also more highly expressed during pathogenic growth was NepA, which belongs to a family of NLP genes. These genes encode effectors required for pathogenicity in some organisms, and recent data show that they also may play diverse roles in the pathogen including development [56]. Oxidative stress response pathways genes were also up-regulated during the early stage of interaction with maize seeds. This is consistent with other plant pathogen interactions in which high oxidative stress conditions are present at the fungus-host interface [57], [58]. Under pathogen attack, ROS metabolism is regulated by a network that involves at least 152 genes in *Arabidopsis* [59]. Fungal pathogens have developed ways to sense and modify ROS accumulation in host plants [60], for example, by the secretion of SOD and CAT, which convert the ROS into less reactive molecules. Consistent with the importance of host ROS in defense against *A. flavus*, we found *A. flavus* antioxidant mutants (Δ*sod*, Δ*ahp*) to be impaired in growth and to produce less aflatoxin than wild type in living seeds. All this poses oxidative stress as a critical factor in the switch from saprobic to pathogenic attitude of *A. flavus* in the interaction with the maize seeds.

Finally, our data show that conidia can carry a memory of the medium from which they were produced. Cultures initiated from these conidia show specific carbon utilization profiles. These results imply that the ability to use carbon is influenced by stored molecules and/or transcripts in the conidium, or perhaps by epigenetic factors. In an ecological setting this could have an advantage by allowing rapid colonization of the substrate in advance of the fungal mycelium. This study provides a genetic and physiological view of different phases in the life cycle of *A. flavus* in maize, and shows the importance of oxidative stress, carbon utilization by differently adapted conidia, and the onset of the secondary metabolism during pathogenicity. Our experimental approach and data analysis provide new insights into the ecology of *A. flavus* and its interaction with maize seeds.

## Materials and Methods

### Fungal strains and culture conditions

Wild type (WT) strain *Aspergillus flavus* NRRL 3357 [used for generating the *A. flavus* complete sequence at the Institute for Genomic Research, Rockville, Maryland (US)], a producer of the aflatoxin B_1_, mutants Δ*sod* [61] and Δ*ahp* (Swartzburg & Payne, unpublished) were incubated on Czapek Dox Agar (CDA) (Difco, USA) for 7 days at 30**°**C, before use. Czapek Dox Broth (CDB) (Difco, USA) was inoculated with the WT or the Δ*sod* and Δ*ahp* mutant strains using 0.1 mL of conidial suspension (∼10^6^ conidia) for each flask; incubation was performed at 30**°**C up to 4 days. This growth condition is designated as *flask* (i.e. the *in vitro* phase). The same quantity of *A. flavus* conidia was also incubated into 100 mL Erlenmeyer flasks containing CDB (50 mL) and a dialysis membrane (MWCO –7000 Da) in which 10 injured viable kernels of maize were placed. This condition is referred to as *chemo* phase (i.e. the chemotrophic phase). The dialysis membrane should allow the passage of small molecules from the injured kernels into the CDB medium. Ten grams of non-viable maize kernels (autoclaved) were inoculated with 10^4^ conidia in a sterile Petri plate and incubated for 4 days kept in a phytotron in which the temperature/RH/light (23_min_−33_max_ **°**C/80–70%/∼16h light) cycles were set similar to those naturally occurring in August in North Carolina (US). This condition is referred to as *sapro* (i.e. the saprophytic phase). Developing maize kernels at the R4 stage were wounded at the top with a needle that had been immersed into a 10^6^ conidial suspension of *A. flavus*. This condition is referred to as *in vivo* (i.e. the pathogenic phase). The mycelia, or the kernels infected with the fungus, were collected after 4 days and used for the subsequent micro-array experiment. To minimize the noise between replicates for the expression analysis, three biological replicates were used for RNA extraction and amplification.

### Fungal growth and aflatoxin production

At each time point, fungal growth on liquid media was determined by weighing the mycelium after filtration (Millipore filters, 0.45 αm) and drying it for 48h at 80**°**C. Fungal growth on both viable and non-viable maize kernels was measured by DNA quantification, using the method described below. Aflatoxin (AF) production was analyzed in culture filtrates of the WT, Δ*sod* and Δ*ahp* strains following extraction with chloroform:methanol (2 ∶1 vol/vol). The extracts were collected; the volume was reduced under a stream of nitrogen; and the quantitative analyses were carried out by HPLC, as previously reported [39].

### DNA extraction and amplification

Total DNA was extracted from 30 mg (dry weight) of freeze-dried maize kernels that were inoculated with *A. flavus,* or from 20 mg (d.w.) of mycelia harvested from *flask* and *chemo* cultures. DNA extraction was performed according to the Farber method [62] slightly modified; in our extraction protocol we used a mixture of phenol–chloroform–isoamyl alcohol (25 ∶24 ∶1) instead of pure phenol for the purification of the sample and isopropanol instead of absolute ethanol to precipitate DNA. The precipitated DNA was washed with 70% ethanol and finally re-suspended in 50 αl of filter-sterilized distilled water.

### Microarray analysis

Data were generated using custom Affymetrix GeneChip microarrays containing 22683 probes (negative and positive controls, *Z. mays* and *A. flavus*). Raw data were normalized in JMP Genomics (SAS Institute Inc, Cary, NC) using the robust multichip average (RMA) [63], [64] algorithm followed by a Lowess normalization. This array was then filtered by species and 13548 probes mapping to *A. flavus* (raw data) were selected. Probes with absent calls were removed to yield a dataset with 10978 probes. All array data were deposited in the Gene Expression Omnibus database as experiment GSE28163. These 10978 probes were tested for differential expression between conditions using the DEDS [30] package for R [65]. DEDS calculates several statistics for differential expression and ranks the genes according to the difference between maximal and actual value for each statistic. We used a synthesis of t-statistic, B-statistic, SAM-statistic and fold change, and applied an FDR threshold of 0.01 for selecting the differentially expressed genes. Using these criteria, 9 genes were differentially expressed between *flask* and *chemo* conditions, and 806 genes were differentially expressed between *in vivo* and *sapro* conditions. The pathway analysis was based on the Z-score [66], which represents the difference in standard deviation between the average expression values of the genes in a gene-set and the average expression of all genes in a sample. The statistical significance of the Z-score was assessed by comparing the observed Z-score to a Z-score distribution obtained from 10,000 random gene selections. The p-value is estimated as the number of random permutations that produce higher scores than the observed one. Gene-set used for the pathway analysis were collected from Gene Ontology [67], InterPro [68] and secondary metabolite clusters [11].

### Co-expression of neighbor genes

In order to evaluate the extents of computationally delineated secondary metabolite clusters, each of the *A. flavus* chromosomes (scaffolds) was searched for the stretches of adjacent co-regulated genes using a modified version of the algorithm described in Zhan et al. [69]. Two genes were considered coexpressed neighbors, if i) they were immediately adjacent on chromosome, or were spaced by at most one other gene, and ii) were co-expressed with Pearson Correlation Coefficient at least 0.5 in the expression dataset composed of 75 microarray chips from 28 conditions [26], and the data from current work.

### Motif analysis

Upstream sequences (1000bp) for all the *A. flavus* genes were retrieved from the Ensembl Fungi website. Known regulatory motifs were searched on the upstream sequences using the RSAT software [70]. The statistical enrichment of a motif in a list of differentially expressed genes was assessed by hypergeometric test.

### Quantification of Fungal Growth by Real Time-PCR

*A. flavus* specific primers for *omtA* (*aflP*) (FOR 5′-GCGTCGTATCAAAGCCTCTT-3′; REV 5′-CCACGAATTCATGTCAACCA-3′) were designed using the Primer Express software 3.0 and were used to amplify a 330bp fragment. Real Time qPCR was prepared in triplicates of 20 μl reaction mixtures containing 50 ng of a mixture of maize and *Aspergillus* DNA, SYBR-Green I Mix 1X (Quantace) and omtA_for/omtA_rev 0.5 αM primers this was a). Real Time-PCR was performed in a LineGene K PCR detection systems (Bioer, Japan) with the following cycling conditions: 95**°**C for 10 min, followed by 35 cycles of 95**°**C for 15 s, 67.1**°**C for 30 s and 72**°**C for 20 s. A standard curve was constructed using the *A. flavus* genomic DNA in a concentration range of 100 ng-1 pg. This curve was then used as a reference standard for extrapolating quantitative information for DNA targets of unknown concentrations. Real Time-PCR amplification reactions were carried out in triplicate from 3 biological replica.

### Validation of a Subset of Differentially Expressed Genes by quantitative PCR

RNA extraction was performed with the TRI REAGENT method (Sigma-Aldrich, USA) modified by adding a purification step with chloroform:isoamylic alcohol (24∶1) followed by RNA precipitation over-night with 8 M LiCl. Total RNA from NRRL3357 mycelia was extracted after 4 days for the liquid cultures (*flask, chemo*) and for the maize seed cultures (*sapro, in vivo*), and was used to develop a relative RT-PCR on the cDNA derived from the genes listed in Table 1 with primers listed in Table S5 (supporting information). RT reactions were performed as described previously [23]. The amplifications were also performed on cDNA derived from uncontaminated maize seeds using the same primers and in the same amplification conditions in order to avoid false amplicons derived from plant mRNA. *A. flavus* α-tubulin was used as housekeeping gene (HKG) to normalize differences in total RNA target input and quality and in RT efficiency (Table S5).

### Biolog Phenotype MicroArray (PM)

Carbon-utilization patterns were investigated using FF MicroPlates (Biolog). The FF MicroPlate test panel comprises 95 wells with different carbon-containing compounds and one well with water. The microplates are produced with test reagents prefilled and dried into the 96 wells with a redox dye. Iodonitrotetrazolium violet (INT) is used as a redox dye to colorimetrically measure mitochondrial activity resulting from oxidation of metabolizable carbon sources. All the wells are colourless when first inoculated. The oxidation of succinate to fumarate in the citric acid cycle, mediated by succinate dehydrogenase and FAD, causes INT to be reduced to a red-coloured formazan dye with peak absorbance at 490 nm. The reduction of INT and production of coloured formazan is irreversible, and the accumulation of formazan measured spectrophotometrically quantitatively reflects the oxidation of the test substrate. The conidia for the inoculum were removed from *A. flavus* strains after conidial maturation (5–8 days) in the four different growing conditions described before (*in vivo, chemo, flask* and *sapro*). Conidia were suspended in 20 ml sterile Phytagel solution [0.25% (w/v) Phytagel, 0.03% (v/v) Tween 40] in disposable glass test tubes provided by Biolog (FF Inoculation fluid). The concentration of spores in the suspension was adjusted to the optical density of 75 (±2)% transmissions at 550 nm wavelength. Each conidial suspension was dispensed in three replicates after vortexing (100 µl of suspension for each well) into the wells of a Biolog FF MicroPlate. Inoculated microplates were incubated in the darkness at 30**°**C, and OD readings determined after 0, 24, 48, 72, 96 and 168 h using a microplate reader (V-max Molecular Device), which measures the optical density at 490nm and 750 wavelength (the colour developed by fungal respiration, and turbidity, which reflects mycelial production on the tested substrate).

The 95 substrates were divided into 15 categories plus water in accordance with Atasanova & Druzhinina [38] and the average absorbance for all wells in each category calculated. The categories are: Water, Heptoses, Hexoses, Pentoses, Sugar acids, Hexosamines, Polyols, Polysaccharides, Oligosaccharides, Glucosides, Peptides, L-amino acids, Biogenic and heterocyclic amines, TCA-cycle intermediates, Aliphatic organic acids, Others. ANOVA, followed by Tukey's HSD t-test, was used to verify the significance of the differences between carbon sources utilisation by the four growth phases (C =  chemo, F =  flask, IV =  in vivo, S =  sapro). Statistically significant differences (p<0.001) in substrate use between the growth phases (C =  chemo, F =  flask, IV =  in vivo, S =  sapro) are marked with different letters (A, B, C, D) in Tables 3 and 4. A black/white gradient is used in the tables to graphically represent the degree of overall use of group-substrate.

### Statistical evaluation of Biolog Data

Analyses of the Phenotype MicroArrays were performed on the colour data sets measured at 490 nm, the values being directly proportional to substrate use. One-way ANOVAs were computed with XLSTAT (Addinsoft, Paris) to determine the significant differences between the substrates utilized by the four cultures of the fungus. Moreover, Agglomerative Hierarchical Clustering (AHC) using Euclidean distance measures of the carbon source utilization profiles after 24, 48, 72 and 96 h of incubation was performed. AHC was used to make up homogeneous groups of objects (classes) on the basis of a matrix describing the similarity between the objects. The resulting dendograms show the progressive grouping of the data. A further multivariate method, Principal Coordinate Analysis (PCaA), was used to visualize in one single plot the dissimilarities of the cultures based on their substrate usage in the Biolog assay. PCaA allowed for the graphical visualization of the square matrix that describes the dissimilarity between the groups. A PCoA applied to the matrix of Euclidean distances between observations (calculated after standardization of the columns using the unbiased standard deviation) leads to the same results as a PCA based on the correlation matrix.

Differences in the growth of *A. flavus* in the different culture conditions are depicted in Table 5. One obvious morphological difference in chemo treatment was the production of hyphal aggregates on the dialysis membrane containing injured maize kernels and sclerotia.

## Supporting Information

Figure S1 **Growth (mg/mL dry weight, d.w.) and AF biosynthesis (ppb) in WT and AfΔ** ***sod*** **mutant inoculated in CD medium amended with CH 1mM, incubated at 30°C after different periods (from 3d to 7d).** The data are the mean of 6 separate experiments ± SE.
(DOCX)

Table S1 **The complete list of 815 genes differentially expressed using an array of 13548 genes analyzed in three biological replicates with four different conditions: AF3357 grown on CD medium (** ***flask*** **), grown in flasks with CD medium containing injured maize kernels within a closed dialysis tube (** ***chemo*** **), grown on autoclaved maize kernels (** ***sapro*** **) and grown on ears in the field (** ***in vivo*** **).** Gene expression was compared as follows: *chemo* vs *flask* and *in vivo* vs *sapro*. Statistically significant differences were assessed by Differential Expression using Distance Summary (DEDS) analysis.
(XLS)

Table S2 **Analysis of changes in expression of predefined biological pathways and gene sets.** Gene sets were obtained from Gene Ontology (1085 GO terms covering 4903 genes) and InterPro annotations (1437 domains covering 4900 genes). The statistical significance of the pathway ‘activation’ has been assessed by random permutations (see Methods for details).
(XLS)

Table S3 **ANOVAs analyses on the use of 95 different carbon sources by cultures of** ***A. flavus*** **started from conidia harvested from different trophic phases (** ***chemo, flask, sapro, in vivo*** **).** The test showed statistically significant differences in carbon source use by the conidia obtained from the four trophic phases of the fungus. Tukey's HSD t-test (p<0.01), was used to verify the significance of the differences.
(XLS)

Table S4 **Identification by the finite state machine algorithm of additional co-expressed genes not identified by the SMURF algorithm.**
(XLS)

Table S5 **List of primers used for cDNA amplification by relative Real time PCR.**
(DOC)
