# Supplementary figures and images for: Genotypic and Phenotypic Versatility of Aspergillus flavus during Maize Exploitation
Source: PLoS One. 2013 Jul 19;8(7):e68735. doi: 10.1371/journal.pone.0068735 (PMC3716879; doi:10.1371/journal.pone.0068735)

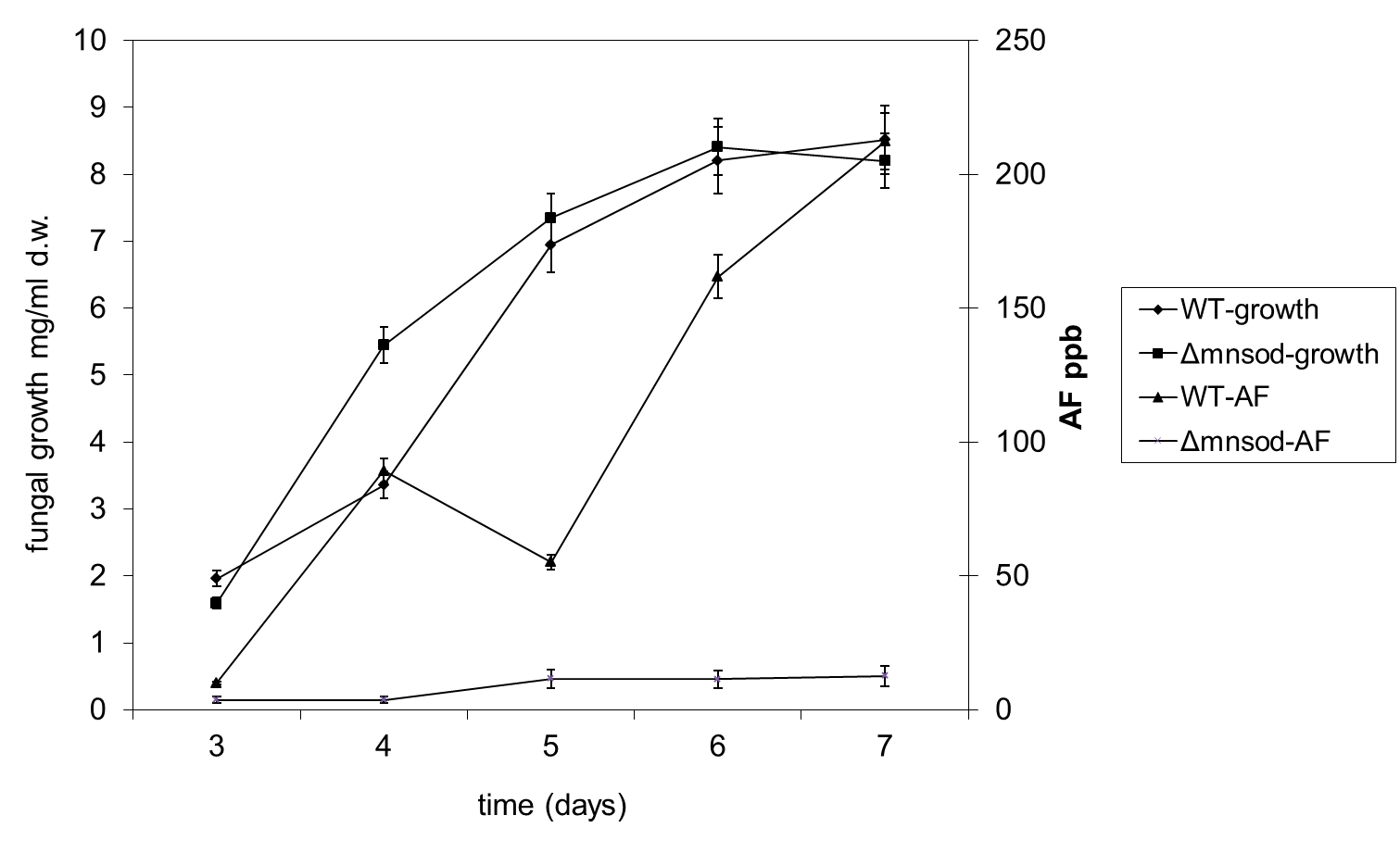


Figure S1. Reverberi et al.

Supplement: Figure S1 — Growth (mg/mL dry weight, d.w.) and AF biosynthesis (ppb) in WT and AfΔ sod mutant inoculated in CD medium amended with CH 1mM, incubated at 30°C after different periods (from 3d to 7d). The data are the mean of 6 separate experiments ± SE. (DOCX) [file pone.0068735.s001.docx]
