# Supplementary material for: Genotypic and Phenotypic Versatility of Aspergillus flavus during Maize Exploitation
Source: PLoS One. 2013 Jul 19;8(7):e68735. doi: 10.1371/journal.pone.0068735 (PMC3716879; doi:10.1371/journal.pone.0068735)
Supplement: Table S5 — List of primers used for cDNA amplification by relative Real time PCR. (DOC) [file pone.0068735.s006.doc]

**Table S5 Primers used for real-time PCR reactions of fungal cDNA**.

| gene | Probe/gene ID | Sequence (5’-3’) | | Ta (°C) | |
| --- | --- | --- | --- | --- | --- |
| Tubulin beta chain | AFLA_051840 | GGAAGTCAGAAGCAGCCATC  GTGACCACCTGTCTCCGTTT | 66 | |  |
| Fungal specific transcription factor domain containing protein | 1918.m01406_at | GCCTCTTTCTCCTCACACG  GGGAATGTTCGTATCGCAGT | 64 | |  |
| Major Facilitator Superfamily protein, membrane transporter | 1918.m01405_at | TCTCCTGGATTCCATCCTTG  TTGACGCCATCATTAGACCA | 64 | |  |
| kelch-domain protein, putative, cell polarity protein | 2911.m00243_at | GCTCTCTACCCCGCTTTTCT  AGAGGAACTCCACAGCCTGA | 64 | |  |
| MED7 protein, transcriptional initiation | 2689.m00650_at | TCAGTAGCCCCTGAGCAGTT  AACCCCGTCCTTCTTCAGTT | 64 | |  |
| GTP binding protein (Gtp1), putative | 2258.m00669_at | TTCGATGTCAGCAAGAGTGG  ATTCCAACACACCCGGAATA | 64 | |  |
| Histidine acid phosphatase family protein | 1918.m01221_at | AGGGTGCAACCAAACTCAAC  TGGTACCGTTGTGGAAGACA | 64 | |  |
| pectin methylesterase, putative | 1866.m00688_at | CTTGAAACCGACACGTCCT  CATACAGACCAGCCAGCAGA | 64 | |  |
| G protein-coupled receptor alpha-related | 1569.m00043_at | TCTCATCACGTGGGTCATGT  CGGTGGTGATATTGCATTGAG | 64 | |  |
| Glutathione-S-transferase | 2842.m00344_at | TGCGACTTCATCAGATGCTC  CCACGATGTTGTGTGTACC | 64 | |  |
| oxidoreductase, short chain dehydrogenase reductase family protein | 1569.m00528_at | TTGTATGATAGTTTGTATGC  CCGTGTATCGTAGCTAGTTC | 64 | |  |
